# Undiagnosed Chronic Granulomatous Disease, *Burkholderia cepacia complex* Pneumonia, and Acquired Hemophagocytic Lymphohistiocytosis: A Deadly Association

**DOI:** 10.1155/2013/874197

**Published:** 2013-08-24

**Authors:** Maxime Maignan, Colin Verdant, Guillaume F. Bouvet, Michael Van Spall, Yves Berthiaume

**Affiliations:** ^1^Institut de Recherches Cliniques de Montréal, Université de Montréal, Montréal, QC, Canada H2W 1R7; ^2^Département de Médecine, Université de Montréal, Montréal, QC, Canada H3T 1J4; ^3^Département de Médecine, Hôpital Sacré Cœur, Université de Montréal, Montréal, QC, Canada H4J 1C5; ^4^Centre de Recherche du Centre hospitalier de l'Université de Montréal, Montréal, QC, Canada H2W 1T8; ^5^Service de Pneumologie, Centre Hospitalier de l'Université de Montréal, Montréal, QC, Canada H2W 1T8

## Abstract

*Background*. Chronic granulomatous disease is a rare inherited disorder of the phagocyte nicotinamide adenine dinucleotide phosphate (NADPH) oxidase. The clinical course of the disease is marked by recurrent infections, including *Burkholderia cepacia complex* infection. *Case Report*. Here we report the case of a 21-year-old male hospitalized for a *Burkholderia cepacia complex* pneumonia. Despite the broad spectrum antibiotic treatment, fever continued and patient's condition worsened. Anemia and thrombocytopenia developed together with hypofibrinogenemia. The patient died of multiple organ dysfunction 17 days after his admission. Autopsy revealed hemophagocytosis, suggesting the diagnosis of acquired hemophagocytic lymphohistiocytosis. DNA analysis showed a deletion in the p47phox gene, confirming the diagnosis of autosomal recessive chronic granulomatous disease. *Discussion*. In addition to chronic granulomatous disease, recent findings have demonstrated that *Burkholderia cepacia complex* can decrease activity of the NADPH oxidase. Interestingly, hemophagocytic lymphohistiocytosis is characterized by an impaired function of the T-cell mediated inflammation which is partly regulated by the NADPH oxidase. Physicians should therefore pay particular attention to this deadly association.

## 1. Introduction

Chronic granulomatous disease (CGD) is an inherited disorder of the phagocyte nicotinamide adenine dinucleotide phosphate (NADPH) oxidase, which results in impaired production of reactive oxygen species and subsequent compromised antimicrobial defenses [1, 2]. CGD accounts for 1.1% of cases of primary immunodeficiency and the prevalence has been estimated to be close to 1/300000 [3]. 

There are different forms of CGD caused by different mutations in the genes coding for the NADPH oxidase protein complex. However, the majority of cases encountered in clinical practice followed an X-linked pattern of inheritance [3] and originated from a defect of gp1-phox gene, either the gene expression or the protein function. This type of CGD is more severe, and diagnosis is usually made within the first year of life because of recurrent infections [3]. The autosomic recessive form of CGD involves genes that encode p22-phox, p67-phox, and p47-phox. This form is less severe, and consequently, the diagnosis is usually made during the second decade of life.

One of the most frequent germs encountered in CGD patients is *Burkholderia cepacia complex *(Bcc). This motile Gram negative bacillus is involved in at least 3% to 7% of pneumonia and in 18% of deaths in CGD [3, 4]. The treatment of Bcc infection is challenging due to antibiotic multiresistant Bcc strains [5, 6]. Bcc is also known to produce several virulence factors such as catalases, hemolysins, proteases, lipases, and some respiratory mucin-binding adhesins [7–9]. Understanding the complex interplay of these mechanisms of adaptation is of great importance to improve the treatment of Bcc infection [10].

We present a patient with undiagnosed CGD suffering from a Bcc pneumonia whose clinical course is marked by the occurrence of hemophagocytic lymphohistiocytosis (HLH), a syndrome characterized by an uncontrolled immune response. This case report describes the deadly potential of this association as well as a potential common pathophysiological pathway between CGD, Bcc, and HLH.

## 2. Case Presentation

A 21-year-old Caucasian male was admitted to our ICU for severe pneumonia. His past medical and family history was unremarkable with no serious or recurrent infections. He was vaccinated properly and never had any adverse reactions. He had been working in a greenhouse but had become unemployed a few months before being admitted. He smoked half a pack a day and drank alcohol only socially. He had no allergies and was not taking any medication. He had not travelled recently and had not been in contact with an ill person.

**Figure 1:**
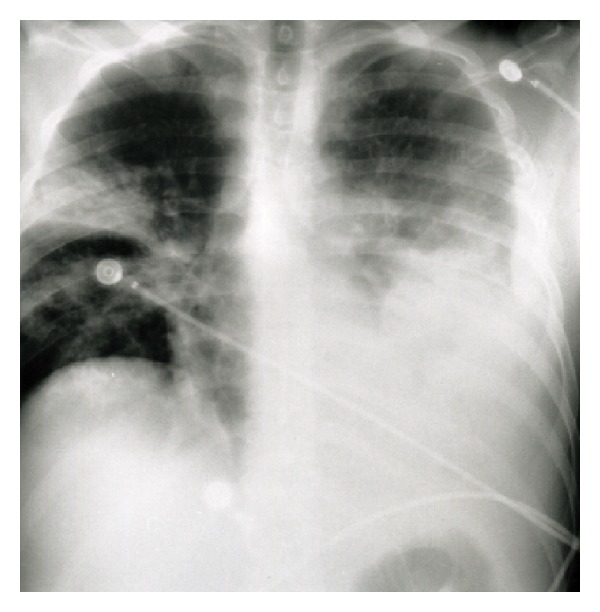
Chest X-ray on the 5th day.

The patient initially had flu-like symptoms and diarrhea. Two weeks after the onset of symptoms, he was admitted to the medical ward for a left lower lobe pneumonia with moderate pleural effusion. Basic laboratory studies showed a white blood cell count of 11,900/mm^3^, with 92% neutrophils, 3% lymphocytes, and no eosinophils. Hemoglobin was 11.5 g/dL and platelet count was 398/mm^3^. Biochemical tests only revealed elevated levels of serum GOT (60UI/L) and GPT (30UI/L). Oxygen and intravenous erythromycin and cefotaxime were started, but cefotaxime was replaced by ciprofloxacin on the 5th day because of a rash. The chest X-ray performed on the 5th day is presented in Figure 1. Blood culture was sterile and a tuberculin shin test was not reactive at 48 hours. Sputum culture revealed Bcc. 

**Figure 2:**
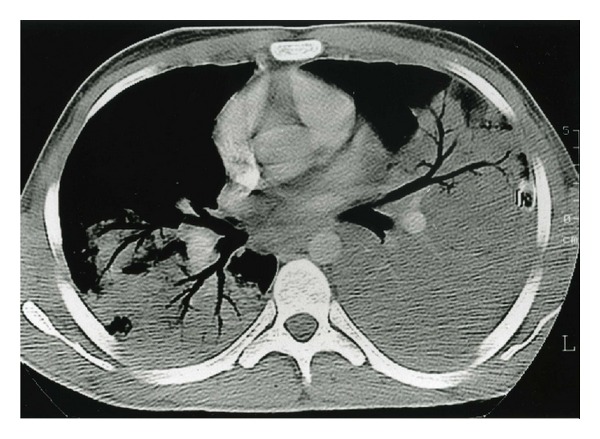
Thoracic CT scan on the 7th day after hospitalization showing a bilateral pneumonia with pleural effusion.

On the 7th day, the patient became severely hypoxemic and was transferred to our ICU. Medical examination and tests showed extensive bilateral pneumonia with increased pleural effusion (Figure 2), splenomegaly, and hepatic cytolysis. The arterial blood gases at 100% oxygen by mask were pH 7.51, PO_2_ 93 mmHg, PCO_2_ 32 mmHg, and HCO_3_ 25 mmol/L. Blood count revealed white-cell count of 4,300/mm^3^, hemoglobin of 88 g/L, and platelet count of 225/mm^3^. Biochemical tests were unchanged. A chest tube was inserted, and piperacillin-tazobactam, netilmicin, and vancomycin were added to ciprofloxacin therapy. Analysis of the effusion yielded pH 7.41, glucose 4.5 mmol/L, LDH 751 UI/L, and protein 33 g/L with cytology negative for tumoral cells.

On the 11th day, the patient was placed on mechanical ventilation because of respiratory failure. The antibiogram showed imipenem and aminoglycoside resistance (Table 1), and antibiotic therapy was switched to ceftazidime and ongoing ciprofloxacin. On the 15th day, hypofibrinogenemia (1.1 g/L), thrombocytopenia (80/mm^3^), and deep anemia (64 g/L) were observed; two units of packed red blood cells were administered. The patient became hypotensive on the 17th day, and despite intensive vasopressor therapy, he died from multiple organ failure, twenty days after hospital admission. An autopsy revealed severe bilateral bronchopneumonia and left empyema associated with hemophagocytosis, prompting the diagnosis of acquired HLH (Table 2). A culture of crushed lung tissues from the autopsy revealed three different species of Bcc. A postmortem DNA analysis showed a deletion (delta GT) in the p47-phox gene, confirming the diagnosis of autosomal recessive CGD. 

## 3. Discussion

Although the association of CGD with Bcc infection is well known, only few case reports have described the presence of HLH [11–14] (Table 3) during the course of Bcc infection in CGD patients. Furthermore, although other medical conditions could lead to infection with Bcc [7], we are not aware of any report of HLH during Bcc infection in absence of CGD. These reports raise the question of a specific interplay between CGD, Bcc, and HLH. Bcc exhibits multiple mechanisms of adaptation, including interference with the phagosome maturation [15, 16]. Moreover, Bcc is able to lessen phagocyte NADPH-oxidase activity, which enables it to survive in phagocyte vacuoles [17]. Thus, in CGD patients, Bcc could further decrease the production of reactive oxidant species by the NADPH oxidase and contribute to the dysregulation of the immune response. 

HLH is characterized by an uncontrolled immune response with severe hyperinflammation [18]. There are two forms of HLH: a familial or genetic one and an acquired one triggered by infections and autoimmune and/or malignant diseases. The clinical course of HLH is mainly characterized by a prolonged fever unresponsive to antibiotics. Patients often present a hepatosplenomegaly and a moderate cytolytic hepatitis. Neurological symptoms as meningism, seizures, and ataxia may be present [19–21]. Adenopathy, edema, or rash may also be encountered, especially in acquired HLH. The diagnosis of HLH has to be made early in the course of the disease in order to initiate potential life-saving treatments with immune modulator treatments such as corticosteroids, cyclosporine A, and methotrexate [22]. This diagnosis is often tricky because of the initial similarity between sepsis and HLH [22]. An eight-criteria panel (Table 2) has been proposed, but diagnosis pitfalls remain [18]. In this case report, the patient fulfilled five of the criteria, which is the threshold for a clinical diagnosis. However, HLH was not diagnosed and the patient did not receive immuno-suppressive drugs.

The pathophysiology of HLH remains controversial, but the common feature of both forms of HLH is an impaired function of natural killer and cytotoxic T cells and their regulatory mechanisms [18]. The secretion of proinflammatory cytokines is increased, while the clearance of pathogens is reduced. Interestingly, recent findings demonstrated that NADPH oxidase plays a crucial role in T-cell mediated inflammation [23]. Human macrophages with the p47-phox mutation exhibit reduced regulatory T-cell induction and T-cell suppression [24]. In CGD patients, the decreased NADPH oxidase activity not only reduced the production of reactive oxidant species and the subsequent pathogens clearance but also impaired the ability of the host to regulate the inflammatory response [1]. Our hypothesis is that the presence of a Bcc infection could further impair the NADPH oxidase activity and, as a consequence, the inflammatory response. Finally, these cooperating mechanisms could explain the association of HLH in CGD patients especially when infected by Bcc.

This pathophysiological cooperation may account for an increased severity. However, there is no cohort study available to investigate the mortality of the CGD, Bcc, and HLH association. We can only state from the published case reports that 2 patients out of 5 died (Table 3). Bcc infection is a recognized severity factor in CGD patients and mortality of infection-associated HLH is close to 20% [3, 25]. The association of these three diseases is likely to have a worse outcome especially in the light of the shared pathophysiological pathways. These considerations may have a strong clinical impact on the general management of CGD patients and on the care of autosomic recessive CGD patients in particular. Autosomic recessive CGD is known to be less severe and to be diagnosed later than the X-linked CGD [4]. Nonetheless, this case report shows that in some autosomic recessive CGD patients, the disease can be revealed by a very severe form of sepsis. The occurrence of HLH in such infected CGD patients may worsen their prognosis especially if CGD and/or HLH are not suspected. 

In conclusion, HLH, CGD, and Bcc infections share pathophysiological pathways, and this possibly explains their relatively frequent association. In light of this case, CGD mutations should be sought out in previously healthy adult patients with Bcc infection, and particular attention should be paid to HLH occurrence during Bcc infections. 

## Figures and Tables

**Table 1:** *In vitro *sensitivity of the 3 types of *Burkholderia cepacia complex* (Bcc) isolated from sputum cultures.

	Minimal inhibitory concentrations (ug/mL) and interpretation		
	Bcc 1	Bcc 2	Bcc 3
Amikacin	>64 R	>64 R	>64 R
Aztreonam	>32 R	>32 R	>32 R
Cefoxitin	>32 R	>32 R	>32 R
Ceftazidime	<8 S	>32 R	>32 R
Ceftizoxime	16 I	128 R	>256 R
Ciprofloxacin	<0.5 S	>4 R	>4 R
Gentamicin	>16 R	>16 R	>16 R
Imipenem	>16 R	>16 R	>16 R
Piperacillin	128 R	>256 R	>256 R
Ticarcillin/clavulanate	>256 R	>256 R	>256 R
Tobramycin	>16 R	>16 R	>16 R
Trimethoprim/sulfamethoxazole	<16 S	<16 S	160 R

S: susceptible; I: intermediate; R: resistant.

**Table 2:** Clinical, laboratory, and autopsy findings corresponding to acquired hemophagocytic lymphohistiocytosis diagnostic criteria.

Hemophagocytic lymphohistiocytosis diagnostic criteria (diagnosis is established by fulfilling 5 out of 8 of the following)	Patient's findings
Fever	Yes
Splenomegaly	Yes
Cytopenia (affecting ≥ 2 cell lineages, hemoglobin < 9 g/dL; platelets < 100 G/L; neutrophils < 1.0 G/L)	Hemoglobin 6.4 g/dL Platelets 80 G/L
Hypertriglyceridemia (≥265 mg/dL) and/or hypofibrinogemia (≤1.5 g/L)	yes
Low or absent natural killer cell cytotoxicity	NI
Hyperferritinemia (>500 ng/mL)	NI
Elevated sCD25 (>2.400 U/mL)	NI
Hemophagocytosis in the bone marrow, spleen, or lymph nodes without malignancy	Bone marrow and spleen

NI: not investigated.

Note: adapted from the 2nd International HLH study, http://www.histio.org/page.aspx?pid=389.

**Table 3:** Chronic granulomatous patients with *Burkholderia cepacia complex* infection and acquired hemophagocytic lymphohistiocytosis.

Sex	Age	Mutation	Site of infection	Outcome	Reference
M	36 months	X-linked	Abdomen	Survived	[11]
F	19 years	p22-phox	Vagina	Survived	[12]
M	17 months	NS	Spleen	Survived	[13]
M	40 months	X-linked	Lung	Deceased	[14]
M	21 years	p47-phox	Lung	Deceased	Present case

NS: non specified.
